# *Malassezia* Folliculitis: An Underdiagnosed Mimicker of Acneiform Eruptions

**DOI:** 10.3390/jof11090662

**Published:** 2025-09-10

**Authors:** Natalia V. Chalupczak, Shari R. Lipner

**Affiliations:** 1 Chicago Medical School, Rosalind Franklin University of Medicine and Science, Chicago, IL 60064, USA; nac4016@med.cornell.edu; 2 Department of Dermatology, Weill Cornell Medicine, New York, NY 10021, USA

**Keywords:** *Malassezia* folliculitis, pityrosporum, review

## Abstract

*Malassezia* folliculitis, previously known as Pityrosporum folliculitis, is a common yet frequently misdiagnosed dermatologic condition caused by *Malassezia* yeast overgrowth in hair follicles. Its monomorphic, pruritic papules and pustules closely mimic acne vulgaris, often leading to inappropriate antibiotic use. This review summarizes current evidence on the epidemiology, clinical presentation, diagnostic challenges, and management of *Malassezia* folliculitis. A high index of clinical suspicion is critical in patients with recalcitrant acneiform eruptions. Diagnosis is supported by dermoscopy, potassium hydroxide preparation, Wood’s lamp, and response to antifungal therapy. Topical and oral antifungal agents are highly effective although relapses are common and access to off-label treatments may be limited. Greater awareness of the distinct clinical features of *Malassezia* folliculitis and treatment response can improve diagnostic accuracy and enhance patient outcomes.

## 1. Introduction

*Malassezia* folliculitis (MF), previously known as Pityrosporum folliculitis, was first described in 1969 [1]. It is a common yet underrecognized skin condition resulting from the overgrowth of lipophilic yeasts of the *Malassezia* genus within hair follicles [2]. MF is often misdiagnosed as acne vulgaris (AV) due to its similar presentation of follicular papules and pustules involving the upper trunk, shoulders, and face [3]. However, unlike AV, MF is typically monomorphic, pruritic, and devoid of comedones. These clinical features raise suspicion for a yeast etiology especially in patients who fail to respond to standard AV treatments [2].

*Malassezia* are lipid-dependent basidiomycetous yeasts (class Malasseziomycetes) with >18 recognized species. In humans, MF is primarily linked to *M. globosa*, *M. restricta*, *M. furfur*, and *M. sympodialis*. Molecularly, *Malassezia* are thought to induce keratinocyte production of inflammatory cytokines (such as interleukin (IL)-lα, IL-6, IL-8, IL-12), tumor necrosis factor (TNF)-α, and anti-inflammatory cytokines IL-4 and IL-10) via Toll-like receptor 2 (TLR 2). *Malassezia* activate complement cascades by both the classic and alternative pathways. Host factors such as humidity, occlusion, antibiotics, and immunosuppression facilitate overgrowth [2].

Despite its high prevalence in warm, humid climates and among immunosuppressed individuals, MF remains underdiagnosed in clinical practice and underrepresented in dermatologic literature. This diagnostic gap frequently leads to inappropriate antibiotic use, delayed initiation of antifungal therapy, and prolonged patient morbidity [4].

This clinical review aims to provide a comprehensive summary of the pathophysiology, clinical presentation, diagnostic strategies, and evidence-based management of MF. By increasing physician awareness and diagnostic accuracy we hope to improve the timely recognition and effective treatment of this frequently misidentified condition.

## 2. Epidemiology

MF is most commonly observed in adolescents and young adults [5] likely due to increased sebaceous gland activity during this period [6]. The frequency and density of *Malassezia* colonization correlates with both age and sebum production [7]. MF is more common in males vs. females [8]. The condition is also more prevalent in individuals living in hot climates likely due to excessive sweating [9]. For example, in a retrospective study of 48 patients with cytology-confirmed MF (mean age of 22.2 years; male to female ratio 11:1; disease duration mean of 10.9 months), 83% reported working in a warm environment and 71% reported that sweating was an aggravating factor. The majority (*n* = 37) reported pruritus and the remainder (*n* = 11) were completely asymptomatic [10].

MF is commonly reported in immunosuppressed populations, including patients with HIV, history of organ transplant, or those receiving corticosteroids [11], which may be due to colonization of *Malassezia furfur* in this patient population. For example, in a cross-sectional study of 185 HIV-positive patients, 25 (13.5%) had *Malassezia* infections, of whom 4 (16%) were clinically diagnosed with MF and most cases (9/25) occurred in patients with AIDS [12]. In addition, in a prospective comparative cross-sectional study of 96 subjects (48 HIV seropositive and 48 HIV seronegative), *Malassezia furfur* was isolated from 16.7% of HIV-seropositive individuals compared to only 1.3% of HIV-seronegative individuals (no *p*-value reported) [13].

## 3. Clinical Presentation and History

**Figure 1:**
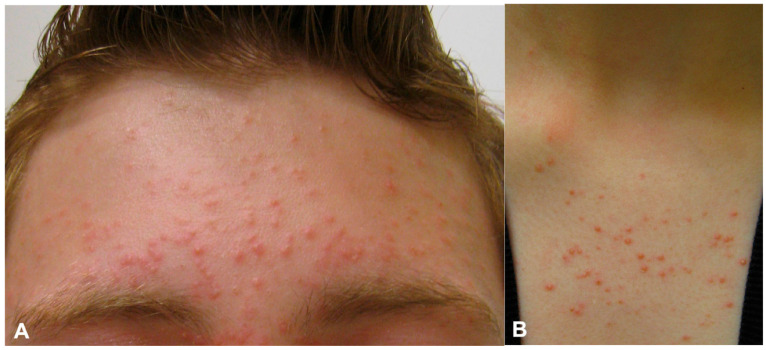
Fine monomorphic papules and pustules on the forehead extending into the hairline with mild associated scaling of the eyebrows (**A**) and the upper chest (**B**).

MF most commonly presents as mildly pruritic, 1–2 mm, monomorphic follicular, erythematous or skin-colored papules and pustules centered around hair follicles and distributed across seborrheic areas such as the upper trunk, chest, back, shoulders, and face (Figure 1) [14]. Excoriations and hyperpigmentation are common. In a prospective study by Durdu et al. [15] assessing lesion distribution in 49 patients with MF, 71.4% had involvement of multiple regions, with the face most often affected (57%), followed by the back (53%), extensor arms (38.8%), chest (36.7%), and neck (18.3%). A hallmark feature distinguishing MF from AV is the absence of comedones, nodules, or cysts [16]. When symptomatic, the most commonly reported symptom is mild to moderate pruritus. For example, in a retrospective cohort study by Prindaville et al. of 110 patients with MF, 65% (*n* = 72) reported pruritus [8]. Additionally, in a prospective cohort study of 55 MF patients, 71% (*n* = 39) experienced pruritus [17].

MF tends to follow a chronic-relapsing course and responds quickly to antifungal treatment with frequent recurrences [2]. Inappropriate or prolonged use of systemic antibiotics or topical corticosteroids may temporarily suppress inflammation, leading to diagnostic confusion followed by rebound flares upon discontinuation [18].

Patients frequently present with a history of failed treatment for presumed AV, sometimes after months of oral antibiotic therapy. For example, in the retrospective study [8] by Prindaville et al. of 110 patients with MF confirmed with potassium hydroxide (KOH) with microscopy, 75% had been previously treated for AV unsuccessfully. Patients presenting with papulopustular lesions, pruritus, resistance to AV medications, and lack of comedones should prompt consideration of MF as a diagnosis. 

## 4. Diagnostic Workup

The diagnosis of MF is primarily clinical, based on characteristic morphology, distribution, and symptoms (Supplementary Figure S1). However, distinguishing MF from AV or bacterial folliculitis can be challenging especially in the absence of pruritus or when it only involves the face. As a result, ancillary testing may be necessary to confirm the diagnosis in cases refractory to conventional AV therapies.

**Figure 2:**
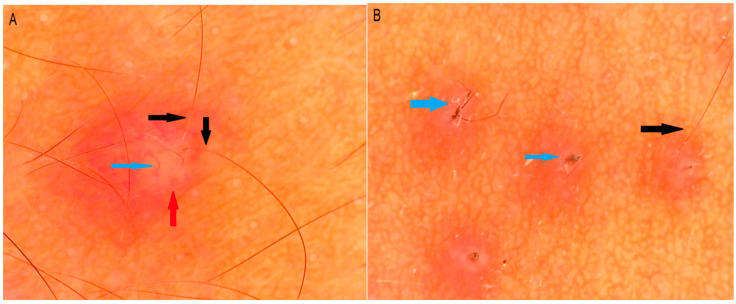
Folliculocentric lesion with surrounding erythem (red arrow), tortuous vessel (blue arrow) and hypopigmentation of the proximal hair shaft (black arrow) (**A**) and broken hairs (blue arrow) and hypopigmentation of proximal hair shaft (black arrow) (**B**). [Dinolite AM4115ZT; 150×; polarizing].

Dermoscopy is a noninvasive tool that may aid in the clinical diagnosis of MF. In a case series of 15 patients with MF analyzed with dermoscopy, scale was the most common feature detected in 11 patients (73.3%) (Figure 2), followed by hypopigmentation of the affected hair shaft in 9 cases (60%), and keratosis pilaris–like features in 8 cases (53.3%). Dermoscopy of resolving lesions was nonspecific, showing only residual brownish discoloration [19]. While its diagnostic role is not yet standardized, dermoscopy can provide supportive findings that complement clinical evaluation.

Wood’s lamp examination can also serve as a useful noninvasive adjunct. Under ultraviolet light (320–400 nm), MF lesions may exhibit yellow-green fluorescence [15]. For example, in the cohort study [15] by Durdu et al. of 49 patients with MF confirmed by cytology, May-Grünwald-Giemsa smear had 100% sensitivity, KOH test 81.6% sensitivity, and Wood’s lamp examination 66.7% sensitivity. Although Wood’s lamp examination is fairly sensitive for MF, it is neither genus nor species specific.

Ultraviolet dermoscopy, which combines the magnification and surface detail of dermoscopy with the enhanced contrast of ultraviolet fluorescence (365 nm), can be used to enhance visualization when diagnosing MF by identifying key features. For example, in a case report [20] describing a 17-year-old female with biopsy-confirmed MF, ultraviolet dermoscopy demonstrated bluish white fluorescence confined to the affected follicles. Additionally, in a multicenter retrospective study [21] evaluating the diagnostic accuracy of ultraviolet-induced fluorescence dermoscopy in non-neoplastic dermatoses, 11 out of 13 MF patients (84.6%) exhibited blue follicle-bound fluorescence (*p* < 0.001), and diagnostic sensitivity and specificity were 84.6% and 100%, respectively (diagnostic odds ratio = 151.8). The bluish follicular fluorescence can be contrasted with acne which usually shows no fluorescence and presents as follicular blackouts (absent cutibacterial reddish fluorescence) [20].

**Figure 3:**
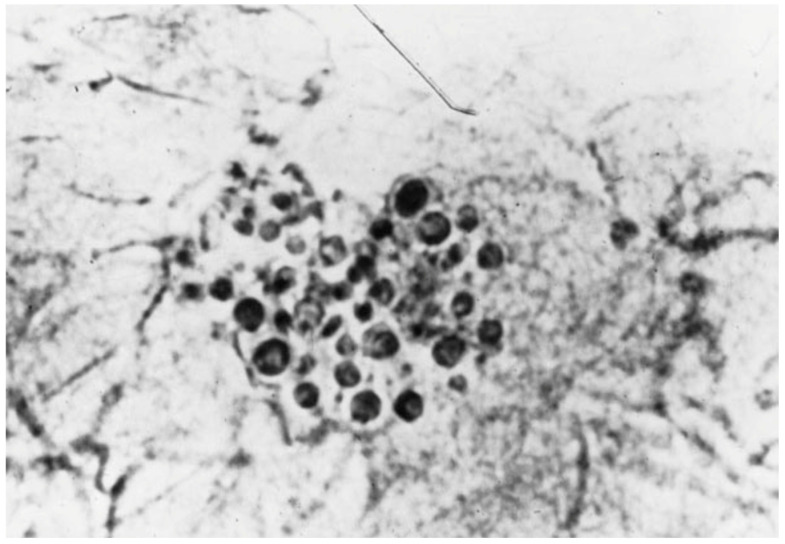
Typical morphology of *Malassezia* species seen in a KOH preparation of skin scrapings, revealing yeastlike bodies and hyphae.

KOH preparation is the most accessible and cost-effective diagnostic tool for diagnosing MF. Direct microscopy is performed on superficial skin scrapings or extraction of follicular contents on a glass slide and dissolved in 10–20% KOH. Visualization may show clusters of round budding yeast and short hyphae (Figure 3) [22,23]. For example, in a case series by Ayers et al. including 6 adolescent women with concurrent MF and AV, KOH examination of skin scrapings of pustules showed numerous spores and budding yeast forms [5]. A positive KOH finding can be diagnostic when clinical suspicion is high. However, *Malassezia* is part of the normal skin flora and present in the stratum corneum of almost all healthy individuals. For example, in a cross-sectional study of 27 healthy neonates, *Malassezia* spp. were detected in 89% of skin samples on day 0 and 100% of the samples on day 1 [24]. Therefore, since *Malassezia* often colonizes the skin, demonstration of presence of *Malassezia* deeper within the hair follicle may provide a more accurate diagnosis of MF.

Fungal cultures are generally not recommended for diagnosis of MF due to the fastidious growth requirements of *Malassezia* species and their status as common skin commensals. In select cases they may be performed using specialized media including Dixon’s, Leeming–Notman agar, and Ushijima’s [25]. Furthermore, cultures showing *Malassezia* species may indicate colonizers rather than pathogens and confound diagnosis. For example, in a cross-sectional study [26] using culture and polymerase chain reaction (PCR) analyzing *Malassezia* species isolated from MF lesions and non-lesional skin of 32 MF patients and 40 healthy controls, respectively, the same predominant species (*M. restricta*, *M. globosa*, *M. sympodialis*) were found across all groups, with no difference in species number or total burden (samples from MF lesions: 1.7 ± 0.7 (mean ± SD) species, normal skin of MF patients: 1.5 ± 0.9 species, normal skin in healthy controls: 1.9 ± 0.8 species (*p* > 0.05)). Therefore, pathogenesis of MF is likely due to overgrowth or dysregulation of resident cutaneous *Malassezia* species rather than caused by exogenous or novel strains. Consequently, it is important to interpret culture results in clinical context.

PCR-based assays are a sensitive and specific method to detect *Malassezia* deoxyribonucleic acid (DNA) directly from lesional material. Unlike microscopy or culture, PCR allows for species-level identification and can detect low fungal burdens. For example, a laboratory-based PCR study of 107 clinical isolates [27] identified 6 *Malassezia* species (*M. restricta*, *M. globosa*, *M. sympodialis*, *M. furfur*, *M. pachydermatis*, and *M. slooffiae*) using species specific primers targeting 26S rDNA sequences with good concordance with conventional DNA sequencing methods. Real-time PCR has a rapid turnaround and can overcome the limitations of culture which is often slow and may miss lipid-dependent species. However, because *Malassezia* is part of the normal skin flora, PCR is unable to confirm pathogenic transformation [22]. Although not yet widely adopted in clinical dermatology due to cost and access, PCR is a promising adjunct for diagnosing MF.

Histopathologic examination via skin biopsy may be the most definitive technique for confirming a diagnosis of MF. Histology typically shows a superficial folliculitis with perifollicular neutrophilic infiltrate and may demonstrate *Malassezia* organisms on periodic acid–Schiff or Grocott-Gomori methenamine silver stains [22,28]. In retrospective study [29] of 52 patients comparing histopathological findings of patients diagnosed with MF and other similar acneiform eruptions, fungal spores in the follicular lumen were most consistent with diagnosis of MF (*p* < 0.001), while acneiform eruptions were more likely to show intrafollicular inflammation (*p* = 0.009), irregular patterns of keratin plugging (*p* = 0.008), and nuclear dust in the follicular lumen (*p* < 0.001).

A 2023 consensus statement [30] by the European Academy of Dermatology and Venerology (EADV) emphasized the importance of considering MF as a diagnosis in patients with persistent or recurrent follicular papules and pustules in seborrheic regions unresponsive to antibiotics. The expert group recommended that diagnostic workup should include clinical examination as well as direct microscopic examination. Bacterial culture may be used when assessing differential diagnoses. If microscopic analysis is unavailable, the expert group supports initiating treatment based on clinical evaluation alone.

## 5. Differential Diagnosis

MF shares overlapping features with several acneiform and follicular disorders which often leads to diagnostic confusion (Table 1). The most frequent misdiagnosis is AV, usually the inflammatory papular subtype [31]. For example, in a retrospective case series by Levy et al. including 26 patients with MF, 65% had been previously misdiagnosed and treated for AV with topical or systemic antibiotics or acne treatments [32]. However, MF is typically monomorphic, pruritic, and devoid of comedones, whereas AV presents with polymorphic lesions including comedones, papules, pustules, and nodules, and is often non-pruritic [31]. Rosacea may share a facial distribution with MF but typically presents with flushing, telangiectasias, background erythema, and papules or pustules, rather than the monomorphic follicular papules seen in MF.

Bacterial folliculitis is another consideration when pustules are present. However, unlike MF, bacterial folliculitis often presents with isolated pustules with surrounding erythema and tenderness, is more localized, and typically responds to antibacterial rather than antifungal therapy [33]. Gram-negative folliculitis, which may develop in patients on long-term antibiotics for AV, can also mimic MF but tends to occur around the perioral area and jawline and is more often pustular and painful [34].

Steroid-induced acneiform eruptions may also mimic MF. Papules often come on quickly and lack pruritus. Large numbers of *Malassezia furfur* have been identified within the hair follicles of patients receiving systemic corticosteroids who developed acneiform eruptions. For example, in a retrospective comparative study [35] of 34 patients clinically diagnosed with steroid acne, 76% (*n* = 26) showed *Malessezia* on direct microscopy. These findings suggest that *Malassezia* species may play a role in the pathogenesis of steroid-induced acneiform eruptions, further complicating clinical differentiation from MF.

A thorough clinical history including symptom chronicity, associated pruritus, prior treatment response, and exposure to antibiotics or corticosteroids combined with physical examination findings is essential for narrowing the differential and guiding appropriate diagnostic testing.

## 6. Management

The literature on the treatment of MF remains limited with most studies including small patient cohorts [15,36]. Therapeutic options and a suggested stepwise approach to treatment are outlined in Table 2 and Supplementary Figure S1, respectively. Supplementary Figure S1 is not a validated algorithm and should be adapted to local resources, clinician judgment, and individual patient factors.

Topical monotherapy with agents such as miconazole, ketoconazole, or econazole has shown variable efficacy. For example, in an open-label comparative study [37] of 51 patients with clinically- and microscopically-confirmed MF evaluating efficacy of selenium disulfide (SeS2) 2% shampoo once weekly (*n* = 25), 50% propylene glycol applied twice daily for 3 weeks (*n* = 12) and econazole cream daily for 1 week followed by once-weekly application (*n* = 10), clinical recovery was observed in 80% (*n* = 20), 75% (*n* = 9), and 70% (*n* = 7) of patients in each group, respectively after 4 weeks (no *p*-value reported). After an additional 4 weeks of continued treatment, efficacy was 88% (*n* = 22), 100% (*n* = 12), and 80% (*n* = 8), respectively (no *p*-value reported). Many patients (no exact *n* reported) experienced relapse following treatment discontinuation, indicating the need for maintenance therapy to prevent recurrence.

Topical monotherapy may be as effective as systemic therapy but with slower response. For example, in a retrospective observational study [38] in Japan including 44 patients with microscopy-confirmed MF with 37 patients treated with 2% ketoconazole cream twice daily and 7 patients treated with 100 mg oral itraconazole daily, clinical improvement (defined as flattening of papules) was achieved after a mean of 27 ± 16 days and 14 ± 4 days, respectively. 

Some studies show that topical treatment may be as effective as oral treatment. For example, in the retrospective study by Prindaville et al. [8] including 110 patients with MF confirmed by KOH, treatment with oral fluconazole (*n* = 16), oral ketoconazole (*n* = 12), and ketoconazole 2% shampoo (*n* = 65) used in combination with cream (*n* = 10), showed 100%, 91%, and 100% improvement rates, respectively.

Both topical and systemic therapy may be effective in immunosuppressed patients. For example, in a case series by Rhie et al. [39] including 11 male orthotopic heart transplant recipients receiving triple immunosuppressive therapy diagnosed with MF confirmed via KOH preparation and/or culture over a 4-month period (mean age 43 ± 9 years), 6 patients responded to topical antifungal therapy (clotrimazole 1% and selenium disulfide lotion) while 5 responded to systemic fluconazole within 3 weeks.

Generally, the most effective treatment is systemic antifungal medication due to the ability to penetrate *Malassezia* in the hair follicle. In severe or recalcitrant cases, oral antifungals offer a more rapid and reliable response. Oral antifungal therapy should be avoided in patients with contraindications including active liver disease, significant hepatic enzyme elevation, history of hepatotoxicity from antifungals, or drug-drug interactions involving hepatic metabolism [5].

Oral itraconazole may be effective for MF. In a randomized, double-blind, parallel group study [40] including 26 patients with MF confirmed by microscopy randomized to itraconazole 200 mg daily or placebo for 7 days, 84.6% (*n* = 11) of itraconazole-treated patients showed negative mycological examination after 5 weeks compared to only 7.7% (*n* = 1) in the placebo-treated group (*p* < 0.01).

Fluconazole has shown effectiveness in several case reports and case series [39] For example, in the case series [5] by Ayers et al. including 6 adolescent females with MF and AV, treatment with oral fluconazole demonstrated positive clinical outcomes in the two patients who were treated. Additionally, in the case series by Rhie et al. including 11 orthotopic heart transplant recipients with biopsy-confirmed MF, 5 patients who failed topical treatment who were subsequently treated with oral fluconazole (100–200 mg/day), showed clinical resolution within 3 weeks. No adverse effects were reported.

Some studies have reported that combination oral and topical therapies are effective for MF. For example, in a prospective interventional study [41] of 62 patients with MF (confirmed histologically and via KOH prep), treatment with systemic ketoconazole alone resulted in clearance in 75% (*n* = 15/20) and improvement in 25% (*n* = 5/20), while combination therapy with systemic and topical ketoconazole showed 100% clearance (*n* = 20/20) (no *p*-value reported). Topical econazole 1% and miconazole 2% were much less effective with no patients achieving full clearance and failure rates of 83% (*n* = 10/12) and 100% (*n* = 10/10), respectively (no *p*-value reported). Among those who initially responded, 25% (no exact *n* reported) experienced recurrence, typically within 4 months. The remaining 75% remained clear at the 6-month follow-up with maintenance with ketoconazole shampoo.

Oral isotretinoin is a retinoid traditionally used for acne that has shown some efficacy in MF in case reports. It may be considered when antifungal therapies are contraindicated or ineffective. Its benefit in MF is indirect and proposed to be via sebosuppression and normalization of follicular keratinization. Three case reports have described the effect of oral isotretinoin treatment in patients with resistant MF. For example, a 23-year-old female with biopsy-confirmed MF treated with 0.65 mg/kg/day of isotretinoin had clinical improvement after 20 weeks [42]. Additionally, a 44-year-old man with biopsy-confirmed MF, resistant to both topical and systemic antifungal therapy (betamethasone valerate 0.1% cream, ketoconazole 2% cream, and oral fluconazole), who was then treated with isotretinoin 60 mg daily for 1 month followed by 90 mg daily for 4 months (average 0.85 mg/kg/day), had complete resolution of MF after 5 months. Four months after discontinuation of therapy, the patient remained clear [43]. Conversely, a 35-year-old man with a 10 year history of eczema and biopsy proven MF, had no clinical improvement in MF with treatment with 1.00 mg/kg/day of isotretinoin for 3 months [44]. Given that isotretinoin does not directly target *Malassezia*, relapse may occur after cessation of therapy [45]. Careful patient selection and monitoring are needed due to its teratogenicity and systemic side effects.

Photodynamic therapy (PDT) involves the application of a photosensitizing agent followed by activation with a specific light wavelength. It has been explored as an alternative treatment for MF. PDT theoretically targets the pilosebaceous unit where *Malassezia* is usually found and may reduce inflammation and fungal colonization. For example, in a pilot study [46] including 6 patients with recalcitrant MF treated with three sessions of methyl 5-amino-levulinic acid (MAL)-PDT at 2-week intervals for 1 month, 50% (*n* = 3) achieved improvement measured by physician assessment. No patients reported any adverse effects except for temporary mild erythema and a stinging sensation during treatment. Device availability and cost vary and treatment requires trained operators and multiple sessions. Long-term safety and durability of PDT in MF remain uncertain. It is important to note that PDT is off-label and best reserved for selected refractory cases. 

Another novel non-pharmacologic treatment modality that has shown promise for treatment of MF is cold atmospheric plasma (CAP) therapy. In a laboratory study [47] conducted by Wang et al., in vitro testing demonstrated that CAP exposure led to a dose-dependent reduction in colony-forming units of planktonic *M. furfur* and *M. globosa* after 1 min of treatment. CAP was also effective against *Malassezia* biofilms which are typically more resistant to standard antifungal therapies. Scanning electron microscopy showed disruption of the extracellular matrix and collapse of the biofilm structure following CAP exposure. Wang et al. then conducted a subsequent randomized control study [47] comparing daily CAP treatment to oral itraconazole (200 mg/day for 2 weeks) in 50 patients with MF. Clinical success (Physician’s Global Assessment score of 0 or 1) was achieved in 40.0% of patients in the CAP group vs. 58.3% in the itraconazole group (*p* = 0.199), with negative direct microscopy rates of 56.0% and 66.7%, respectively (*p* = 0.444). CAP was well tolerated with only mild local side effects (dryness, pruritus, erythema) in 3 patients in the CAP group and 1 patient in the itraconazole group. Access to CAP platforms may be limited and cost prohibitive and treatment protocols are not yet standardized. Physicians should monitor patients that opt for this treatment for irritation and post-inflammatory dyspigmentation. Given that long-term safety and effectiveness data in MF are lacking, CAP therapy should be considered only in refractory cases. 

In the 2023 expert consensus statement [30], the EADV proposed updated recommendations for the diagnosis and treatment of MF. Given that some studies report 100% cure rates with topical antifungals and minimal adverse events, a two- to four-week therapeutic trial aimed at significant improvement rather than complete resolution may be a practical approach. For immunocompetent individuals, topical antifungals such as azoles, ciclopirox, propylene glycol, zinc, or selenium disulfide are recommended as first-line therapy. For immunocompromised patients, the expert group recommends systemic agents such as itraconazole and fluconazole. Recommended prophylaxis therapy is a continuation of the same topical therapies applied less frequently than when treating active disease.

### Treatment Challenged and Considerations

Inappropriate or delayed treatment due to misdiagnosis is common. For example, in the retrospective case series by Levy et al. [32] of 26 patients with MF (22 men, 4 women; mean age 46 years; 5 immunocompromised), among immunocompetent individuals, the average duration of disease prior to diagnosis was 61 months, suggesting a high frequency of misdiagnosis. Sixty-five percent of patients (*n* = 17) had received ineffective treatments with topical or systemic antibiotics or other anti-AV medications. Topical ketoconazole alone led to resolution in 12% (*n* = 3) of cases and oral ketoconazole (used either alone or in combination with topical therapy) was effective in 75% (*n* = 19) of patients. 

Notably, systemic antibiotics are almost always ineffective and may worsen MF by disrupting the normal skin microbiota and promoting yeast overgrowth [4]. Topical corticosteroids may provide transient relief but typically result in rebound flaring upon discontinuation and should be avoided unless part of a short-term combination regimen. The application of topical corticosteroids to active MF can also alter the morphologic appearance of lesions and may promote misdiagnosis. For example, in a case report [18] of a man with biopsy-diagnosed MF, application of corticosteroid cream altered the morphology of his skin lesions and resulted in areas of post-inflammatory hyperpigmentation with flattened or completely resolved follicular papules.

In most cases, prompt initiation of antifungal therapy leads to significant clinical improvement within 2–4 weeks [48]. When treatment fails, physicians should reassess for alternative diagnoses, nonadherence, or the need for systemic therapy.

## 7. Special Considerations

### 7.1. Recurrence and Maintenance Strategies

To date, no studies have directly compared different maintenance treatment strategies for MF. Patient education is an important aspect of preventing recurrence, including emphasizing the role of sweat, skin occlusion, and inappropriate antibiotic use in the possibility of recurrent MF.

Long-term maintenance therapy may be required to prevent recurrence. Maintenance can include intermittent topical antifungal use or continued modification of aggravating factors [49,50]. For example, in the case series by Ayers et al. [5] including 6 female adolescents with concurrent MF and AV, 5 of the 6 patients who responded to oral antifungal treatment required maintenance therapy with ketoconazole shampoo or selenium disulfide shampoo. In some patients, combining topical antifungals with treatments that provide keratolytic effects, such as selenium disulfide or propylene glycol, was effective [23].

### 7.2. Skin of Color Patients

While MF affects all racial and ethnic groups, it may be more difficult to diagnose in individuals with skin of color due to post inflammatory hyperpigmentation obscuring erythema and leading to misclassification as follicular eczema, AV, or keratosis pilaris [9,51]. There is very limited data on MF presentation and treatment in skin of color populations. Beyond small series, most published data consist of isolated case reports, representing an important gap in the literature. Diagnostic weight should shift toward pruritus, monomorphic follicular papules/pustules in seborrheic distributions (upper back, chest, hairline), and the absence of comedones (favoring MF over acne). Cross-polarized or UV dermoscopy can accentuate folliculocentric change when erythema is subtle. 

### 7.3. Immunosuppressed Populations

Patients with HIV, solid organ transplants, hematologic malignancies, or those receiving systemic immunosuppressants may be at increased risk for MF due to impaired cutaneous immune surveillance [52,53]. Although *Malassezia* species are a normal component of the skin microbiome, during states of immunocompromise or antibiotic use, normal skin flora may be disrupted leading to overgrowth of fungal *Malassezia* within the hair follicle. For example, a systematic review [11] including 50 patients with MF (mean age of 38 years, 86.8% male) found that the diagnosis of MF followed initiation of immunosuppressive therapy or onset of an immunocompromised state in 94.1% of patients. Immunocompromised patients had high response rates to antifungals, with MF symptoms improving in 16 of 18 (88.9%) patients treated with oral antifungals, 12 of 13 (92.3%) patients treated with topicals, and 6 of 6 (100.0%) patients treated for underlying conditions. A high index of suspicion for MF in this group is needed when conventional AV treatments fail or when disease onset coincides with initiation of immunosuppressive therapy.

## 8. Conclusions

MF is a frequently overlooked cause of acneiform eruptions. It is often found in adolescents and young adults who present with pruritic, treatment-resistant, papules and pustules. Its clinical similarity to AV may lead to misdiagnosis and inappropriate antibiotic use which delays the initiation of effective antifungal treatment. Recognition of key distinguishing features such as monomorphic lesions, absence of comedones, and pruritus can aid physicians in making an accurate diagnosis.

While response to antifungal therapy is usually rapid and complete, recurrence is common, supporting a need for ongoing maintenance therapies and continued patient education. Underrepresentation of skin of color populations and disease burden among immunosuppressed patients are areas that require further research.

Ultimately, increased clinical awareness combined with careful diagnostic workup can lead to improved outcomes for patients with this underappreciated condition. Further research is needed that prioritizes standardized diagnostic criteria, prospective treatment comparisons as well as more studies including diverse and immunosuppressed patients to optimize care for those affected by MF.

## Figures and Tables

**Table 1:** Clinical and diagnostic features: *Malassezia* folliculitis vs. acne vulgaris vs. bacterial folliculitis.

Feature	*Malassezia* Folliculitis	Acne Vulgaris	Bacterial Folliculitis
Lesion Morphology	Monomorphic papules/pustules	Polymorphic; includes comedones	Erythematous pustules
Pruritus	Common and often intense	Mild or absent	Mild to moderate
Comedones	Absent	Present	Absent
Response to Treatment	Antifungals effective; antibiotics ineffective	Improves with standard acne therapy	Rapid response to antibiotics
KOH Prep	Positive for yeast and short hyphae	Negative	Negative
Wood’s Lamp	May fluoresce yellow-green (non-specific)	No fluorescence	No fluorescence
Distribution	Upper trunk, shoulders, face (seborrheic areas)	Face, chest, back	Often localized (scalp, legs)
Histology	Yeast in follicles on PAS/GMS stains	Follicular plugging, inflammation	Neutrophilic infiltrate in follicles

**Table 2:** Treatment modalities for *Malassezia* folliculitis: dose, duration, and level of evidence.

Therapy Type	Agent/Example	Dose and Duration	Level of Evidence	Notes
Oral antifungals	Itraconazole; fluconazole	200 mg/day for 7–14 days; 200 mg/day for 1–2 weeks	Ib–IIb; III	RCTs and controlled studies support use in moderate-to-severe MF
Topical antifungals	Econazole cream; 2% ketoconazole cream	Daily for 1 week followed by once-weekly application; twice daily for 1–1.5 months	IIb; III	Effective in mild cases, slower onset, adjunct use
Keratolytics	Selenium disulfide; 50% propylene glycol	2% shampoo once weekly for 3 weeks; twice daily for 3 weeks	IIb	Often adjunctive, limited studies
Oral retinoids	Isotretinoin	0.65–1.00 mg/kg/day for 3–4 months	III	Used for refractory disease, not antifungal
Photodynamic therapy (PDT)	MAL-PDT	Three sessions at 2-week intervals for 1 month	III	Limited case reports suggest benefit
Cold atmospheric plasma (CAP)	CAP device	Daily for 2 weeks	IIb	One comparative study, novel anti-biofilm mechanism
Maintenance	Ketoconazole/ciclopirox shampoo	1–2x/week indefinitely	IV	Based on clinical experience and recurrence patterns

Levels of evidence based on Shekelle et al.: Ia = meta-analysis of RCTs, Ib = at least one RCT, IIa = controlled study without randomization, IIb = quasi-experimental study, III = non-experimental descriptive studies, IV = expert opinion.

## Data Availability

No new data were created or analyzed in this study. Data sharing is not applicable to this article.

## Supplementary Materials

The following supporting information can be downloaded at https://www.mdpi.com/article/10.3390/jof11090662/s1, Figure S1: Stepwise approach to the diagnosis and management of *Malassezia* folliculitis.

## Abbreviations

The following abbreviations are used in this manuscript:

MF	*Malassezia* folliculitis
AV	Acne vulgaris
KOH	Potassium hydroxide
PDT	Photodynamic therapy
CAP	Cold atmospheric plasma
PCR	Polymerase chain reaction
UV	Ultraviolet
RCT	Randomized controlled trial
PAS	Periodic acid–Schiff
