# Supplementary material for: Malassezia Folliculitis: An Underdiagnosed Mimicker of Acneiform Eruptions
Source: J Fungi (Basel). 2025 Sep 10;11(9):662. doi: 10.3390/jof11090662 (PMC12471122; doi:10.3390/jof11090662)
Supplement: Supplementary file 1 [file jof-11-00662-s001.zip › jof-3813145-supplementary.pdf]

Supplementary Figure 1: Stepwise Approach to the Diagnosis and Management of Pityrosporum Folliculitis

Step 1: Clinical Suspicion
Monomorphic papules/pustules
Pruritic
Absence of comedones
Recacitrant to acne therapies

↓

Step 2: Diagnostic Workup
KOH prep
Dermoscopy or UV dermoscopy
Wood's lamp
PCR or biopsy (in refractory or unclear cases)

↓

Step 3: Initial Treatment
Mild-moderate: Topical antifungals +/- keratolytics
Moderate-severe or recalcitrant: Oral antifungals +/-topical agents
Immunocompromised: Start with oral antifungals
Contraindications to antifungals: Consider isotretinoin, PDT

↓

Step 4: Maintenance and Education
Intermittent topical antifungal use
Address occlusion/sweat/hygiene
Educate about recurrence and triggers

↓

Step 5: Reassessment if no improvement
Re-evaluate for non-adherence, misdiagnosis, or fungal resistance
